# English Teachers’ Conceptualizations of Key Competencies in English Language Teaching and Challenges to Their Implementation

**DOI:** 10.3389/fpsyg.2022.849099

**Published:** 2022-04-27

**Authors:** Huashan Lu, Changgang Jiang, Fei Guo

**Affiliations:** School of Foreign Languages, Qingdao Agricultural University, Qingdao, China

**Keywords:** English curriculum, junior middle school English teachers, key competencies in English learning, language ability, cultural awareness, learning ability, thinking skills

## Abstract

Limited empirical research on the key competencies of front-line English teachers, particularly primary and middle school English teachers in rural regions, exists in the current literature. This research focuses on front-line instructors and examines their mastery of the key competencies in English language teaching. Through in-depth interviews, this paper examined eight rural junior middle school English teachers on their understanding of developing students’ key competencies in English language teaching, as well as the obstacles and challenges they experienced in curriculum implementation. The study found that although front-line teachers have a positive attitude toward the key competencies, there is still a certain gap between their comprehension of key competencies and policy requirements and practical demands. In order to implement core qualities and effectively promote curriculum reform, we need to strengthen teacher development training for front-line English teachers, especially for primary and secondary school English teachers in rural areas, to improve their understanding and professionalization of the new round of curriculum reform.

## Introduction

In 2014, the Ministry of Education issued “Opinions on Comprehensively Deepening Curriculum Reform and Implementing the Fundamental Mission of Establishing Virtue.” Under this reform, the Ministry proposed to study and formulate a system of key competencies that will boost student development. In September 2016, the framework for developing key competencies in Chinese students was officially released (Lin, 2016). In 2017, the “General High School English Curriculum Standards (2017 Edition)” was promulgated, marking the launch of a new curriculum reform emphasizing the development of key competencies of students. This new standard uses the Key Competencies throughout the entire careers of teachers to help them pursue sustainable development. This new version of the curriculum focuses on the development of key competencies, such as language ability, cultural awareness, thinking skills and learning ability. However, the definition of these key competencies is still expressed in quite general terms (Lin, 2012); it remains a challenge for teachers to implement them (Shi, 2014). Current studies related to the basic education stage mainly focus on the connotation, theory, curriculum, and other macro issues of key competences in English subject (Mei and Wang, 2018a), as well as English classroom practice and evaluation (Cheng, 2017).

In analyzing the professional standards of teachers in China, we have drawn up an outline of “what English teachers be able to teach.” However, what the front-line teachers are most concerned about is the relationship between key competencies and curriculum standards, specifically, what impact the teaching of key competencies will have on the curriculum and the methods by which they should be taught (Shi, 2014). Therefore, the implementation of the “key competencies” in English language teaching in all aspects of the development of the curriculum and how they should be taught is the focus of the current discussion in the English education community. In the literature, limited empirical research exists on the key competencies of English teachers, especially those primary and middle school English teachers in rural areas. Thus, the present research focuses on examining the rural front-line English teachers’ conceptualizations of the key competencies in English language teaching and the obstacles and challenges they might experience in implementing them in their English teaching. The results of this research should help policymakers and education managers in formulating recommendations for implementing the key competencies in English language teaching into the curriculum design.

## Materials and Methods

### Research Questions

This study aims to answer the following two questions:

(1) How do front-line English teachers in compulsory education conceptualize the key competencies in their English teaching?

(2) What are the difficulties and challenges that front-line English teachers may experience in implementing the key competencies in their classes?

### Respondents

This study included 8 English teachers in a rural middle school in Eastern China (Table 1), including 1 school leader engaged in English teaching and 7 front-line teachers. These 8 teachers are responsible for the teaching of English to 28 normal classes in grades 7–9 at the school. All of the participants were female, and 7 of them had teaching experience of more than 5 years.

**TABLE 1:** Demographics of participating teachers.

Name code	Gender	Age (in years)	Seniority (in years)	Education level
T1	Female	51	28	Undergraduate
T2	Female	45	23	Undergraduate
T3	Female	44	20	Undergraduate
T4	Female	50	26	Undergraduate
T5	Female	41	19	Undergraduate
T6	Female	33	11	Undergraduate
T7	Female	30	6	Undergraduate
T8	Female	25	3	Undergraduate

### Data Collection

This study was conducted using a combination of semi-structured interviews (see Appendix) and classroom observations. First, the background, purpose, and core issues of the interviews were explained to the 8 participating English teachers. Based on the connotation of the four domains and specific indicators of the key competencies of English subject (Cheng and Zhao, 2016; Mei and Wang, 2018a,b; Ministry of Education of the People’s Republic of China [MoE], 2018), the participants were invited to do an interview. Each interview outline contained 10–12 open-ended questions, such as “What competencies do you think should be cultivated in Middle School students?” and “What do you think language ability mainly includes?” Each interview lasted approximately 30 min and was audio-recorded. The total duration of the 8 interview recordings was 4 h, 8 min, and 34 s. The researchers transcribed and proofread the recorded interviews to obtain the interview text data of each interviewee. The transcript of the interview was consisted of 76,384 words. Secondly, this study also conducted classroom observations based on individual interviews in order to gain a deeper understanding of the foreign language teaching methods used by the teachers and their efficacy in the classroom.

### Data Analysis

The interview data were analyzed in an inductive manner by identifying categories and coding them (Creswell, 2007). First, the interview texts of each interviewee were divided into different categories based on the interview questions. For example, if the interviewee mentions concepts such as “vocabulary, grammar, and pronunciation” when talking about language ability, they are classified as “language knowledge.” Two researchers read the interview data line-by-line and independently labeled the concepts and categories involved in the key competencies. In order to eliminate the differences between the two researchers with regard to concepts and category recognition, the third researcher checked the annotations, re-read the data to confirm that the concepts and categories accurately represented the interviewee’s cognitive situation and explored how the concepts and categories were related.

## Findings

### Front-Line Teachers’ Conceptualization of the Key Competencies in Their English Teaching

This research first presents the front-line teachers’ conceptualization of the key competencies in their English teaching, which is specifically expressed in four aspects: language ability, cultural awareness, thinking skills, and learning ability.

#### Language Ability

Our findings suggest that 6 of the teachers equated language ability with listening, speaking, reading and writing. A teacher with 23 years of teaching experience stated:

*Key competencies, I think, are to develop students’ ability to speak, read, write, and comprehensively use language, which is communicative ability. To learn is to apply. Learning is not for learning alone, but to use the language in an appropriate way. (T2)*

Our findings also showed that front-line teachers still believe that English classes should focus on learning vocabulary and practicing grammar. Although all the teachers’ paid attention to explanation, memory and repetition in vocabulary and grammar teaching, they ignored the practical uses of vocabulary and grammar. For example, a teacher with 7 years of teaching experience commented:

*In class, I mainly teach students some key points, such as some keywords and sentences, including the grammar knowledge, because in Grade Eight, sometimes it is necessary to re-explain the children these words. Some students even cannot understand the usage of the definite articles and pronouns. (T7)*

It was found that the majority of the teachers were unfamiliar with the concept of discourse and pragmatics because they did not really understand their meaning. This is verified by their self-report in that some pragmatic features, such as “context,” “pragmatic,” “language use,” and “discourse” are rarely used in their syllabus and their teaching practice in class. As one teacher said:

*In my view, “discourse knowledge” refers to the examples provided by our textbooks. They are the knowledge presented in the text, whereas “pragmatic knowledge” is a context of language use in its environment. (T6)*

Above all, the findings showed that some teachers had a broad understanding of discourse and pragmatic concepts, but failed to combine the learning of English language with the cultivation of language skills at a more detailed level. In general, there is still a considerable gap between some English teachers’ conceptualization of language ability and teaching concepts and the cultivation of students’ key competencies in English learning.

#### Cultural Awareness

As Kramsch (2014) states, cultural awareness facilitates second language acquisition. In addition to grammatical rules, there still exist rules for language usage, language habits, ways of thinking and values. What is viewed as decent social behavior in one culture may be quite improper in another. Due to ignorance of the cultural knowledge as well as the cultural background of the target language, students are inclined to apply their way of speaking their native language to the target language. In general, most teachers in the school are able to pay attention to students’ ability to understand the cultural and social phenomena of different countries in the cultivation of students’ cultural awareness. However, front-line English teachers’ understanding of cultural awareness can be mainly manifested in the following three points: (1) The inability to integrate the related cultural knowledge with language skills, and understand the cultures and social phenomena of different countries; (2) The method of comparing and contrasting is overused; (3) Confusing cultural awareness with moral education.

First, the English Curriculum Standards demand that English teachers have the ability to compare, generalize, explain and evaluate the cross-cultural phenomena presented in the textbook. However, the findings from the interview show that middle school English instructors in rural regions lack the ability to integrate language skills with cultural knowledge. So, it is unlikely that present teaching practices will improve students’ perception of culture on the basis of their language skills. For example, a teacher may separate training in language skills from developing cultural awareness:

*In the classroom, I think that learning a language is mainly to cultivate students’ ability to listen, speak, read, write, and use language. In terms of the ability to use language, these qualities and sensibility to culture, language expression, and ability to master and control also need to be enhanced further. (T3)*

Secondly, with regard to finding the differences between English and Chinese expressions, students should be encouraged to appreciate the differences between Chinese and Western cultures. Such comparisons also help to develop their patriotism and global vision. But the method of comparing and contrasting is overused, and other methods such as immersion teaching, content-based approach, or a situational approach are neglected.

*Let the students feel the difference in language. Through these differences, students also realized their feelings about home and sensibility to culture, that is, other languages must be respected. Of course, one must learn his own mother tongue, too. It is to allow students to feel this kind of thing, and it reflects some customs and habits of this society through language for some things that even involve the political economy, human relations, culture, customs, etc., so that students can feel this culture and expand students’ horizons. (T4)*

*We also mentioned the Chinese culture just now. This is an era of globalization, which requires us to know both Western cultures and our own cultures. More importantly, we should see our own advantages. At the same time, we need to learn some good things from other countries. It is very important to make up for our shortcomings through learning the strength from the West. Students should be provided with a global vision by learning a foreign language. (T5)*

Third, teachers find it challenging to incorporate cultural awareness into their classroom teaching and some teachers confuse cultural awareness with moral education. This can be observed in the comments from teachers T1 and T6:

*In terms of cultural awareness, for example, when our students have just learned Mickey Mouse and Donald Duck, teachers can ask questions such as “do you want to be Mickey?” or “Which aspect of Mickey or Donald is most worthy of your study?” The students point out that they can face any difficulties and are willing to help others. These two excellent qualities are worth learning. In each unit, there is an article that can reflect moral education. (T1)*

*A text in book 3 is about the Dragon Boat Race, I mean the Dragon Boat Festival. We often compare it with some festivals in various countries, such as Christmas, Thanksgiving, and Halloween. Through learning these cultural traditions, students will have a comprehensive understanding of them, which helps students to cultivate their national identity and to show their respect to the other countries. This, of course, can broaden students’ international horizons. (T6)*

It is worth noting that most teachers pay attention to the development of intercultural awareness and intercultural communication skills. Students can be encouraged to appreciate the differences between Chinese and Western cultures by comparing the differences in English and Chinese expressions. Such a comparison will also help in improving the students’ patriotism and global vision. Therefore, English teachers need to have the ability to compare, generalize, explain and evaluate the cross-cultural phenomena presented in their textbooks. Suggestions for improving the teachers’ professional ability to increase their students’ cultural awareness can be given, such as bridging the culture gap with good communication skills, celebrating traditional holidays, festivals, and food, and preparing for global citizenship, etc.

#### Thinking Skills

The data from the interviews showed that the teachers’ awareness of the students’ ability to think is less than their language ability and cultural awareness. In practice, teachers encounter problems with the following: (1) there is still no effective method for training students to understand English concepts; (2) nor is there any method to help students to broadly understand thinking skills, for example, to make distinctions, form questions, generalize, expand and use dialectical thinking.

Some teachers pay attention to the different ways of thinking when using English and Chinese expressions and believe that English learning helps students learn to think and solve problems from different angles. For example, one teacher said:

*When we use language, our thinking is different. Chinese and English have different sentence structures. This provides students another way of thinking, which can help students to change the way they look at things and, thus, to be more open and tolerant. (T8)*

However, several teachers confused the ability to think with the development of cultural ethics. For instance, one teacher argued:

*I think “thinking skills” means a feeling of the home country like you mentioned just now. Moreover, some articles concern how children can be independent, which is beneficial for those children’s development. For example, there is one unit, where presents a student’s story of how to be a volunteer in public areas such as hospitals. More voluntary devotions should be advocated in education. (T5)*

Only one of the eight teachers interviewed talked about thinking skills more concretely, but she confused thinking skills with training in learning ability a certain extent.

*I believe “learning ability,” first of all, refers to the ability to learn to listen and to think. Your overall abilities, including the ability to distinguish, question, induct, will promote your growth and development. (T2)*

These findings indicate that it is necessary to strengthen the teachers’ understanding of the ability to think and to provide practical teaching guidance are urgent problems that experts and education management departments need to solve. Firstly, teachers should demonstrate thinking skills to their students, such as sharing creativity, imagination, and thinking skills with the students. The teachers’ own thinking and learning skills should be used to add to the discussions in the classroom and encourage students to share their own thinking. Secondly, through asking metacognitive questions, teachers will enable their students to have a better understanding of the learning process, as well as being able to self-reflect as learners. Example questions may include: Why did you choose to do it that way? When you find something tricky, what helps you? Thirdly, classroom debates can help teachers spark a lively conversation and naturally embed critical-thinking skills by asking students to formulate and support their own opinions and consider and respond to opposing viewpoints.

Critical thinking has the power to launch students on unforgettable learning experiences while helping them develop new habits of thought, reflection, and inquiry. Developing these skills prepares students to examine issues of power and promote transformative change in the world around them.

#### Learning Ability

The interview data show that most of the teachers focus on developing students’ English learning attitudes, interests, and motivations, and students’ awareness and habits of actively participating in language practice. However, these are not enough to develop students’ learning methods and strategies, autonomous learning and sustainable learning attention. English teachers appreciate that English is highly dependent on prior understanding and skill development. As a result, they are most likely to question the readiness of students for grade-level content when the school year begins. Teachers have frequently expressed this point, for example:

*Cultivating students’ interest in learning is mainly through competition and praise. For example, in class, an activity of examination who writes well and who speaks well can be conducted. Sometimes we can conduct a game of word king competition or writing to improve students’ learning efficiency. Also, we can teach dialogues through broadcasting cartoons. Students like to learn English in that way. The habit of learning in junior high school is to allow students to persevere in memorizing every day. First, they have to speak the language, and then read it aloud, and read it aloud every morning for 15 minutes. (T5)*

When teachers have to teach students who lack the necessary skills to learn advanced new concepts, words and structures, there is a constant pressure to build conceptual knowledge through new approaches. Training the students in learning ability and strategies helps teachers to build the students’ confidence so that they can carry out their learning tasks effectively.

### Challenges the Teachers Experienced in Implementing the Key Competencies in Class

This study also investigated the obstacles and challenges that those teachers might meet with in implementing the key competencies of English in their classes. For teachers, the difficulty in implementing the key competencies of English into the course design is mainly reflected in the course content and teaching methods.

#### Course Content

“General High School English Curriculum Standards (2017 Edition)” proposes that the course content should consist of six elements: topic and context, discourse type, language knowledge, cultural knowledge, language skills and learning strategies. It is different from the “English Curriculum Standards (Experiment)” promulgated in 2003 as it emphasizes subject context and different types of text type (Mei and Wang, 2018a). The survey results show that the teachers basically equate the “topic and context” and the “discourse type” with the genre, such as narrative, explanatory text, and discussion paper. A teacher with more than 20 years of experience said the following:

*The “topic and context “is basically based on the topic involved in each unit. For example, in the first unit, how do you spend your holiday during the summer vacation, and where do you take your holiday? The practice surrounding this part is mainly the general past tense. Another example is that you saw something meaningful and interesting on holiday. Generally speaking, one unit talks about one topic. However, relatively speaking, the topic is too difficult for middle and primary school students in rural areas. For these rural children, it is difficult for them to learn English for a lack of a good language learning environment. (T4)*

Since the current junior middle school English textbooks do not have specific training requirements for different types of text, the main content is a daily narrative from the perspective of the contents of the seventh grade to the ninth grade of “Junior Middle School English (People’s Education Edition).” One of the teachers commented as follows:

*Regarding the discourse type, our English textbooks in junior high school have not emphasized this one. Perhaps high school can emphasize this. The content of the textbook is mainly daily narratives and short stories. The topic is basically a daily topic. For example, the first unit is to narrate the holiday story, and the second unit is to ask the students how many times they exercise daily. The third unit says that students introduce their own appearance, each other’s appearance, and learning content to the students’ daily life. (T3)*

Thus, it is necessary to strengthen the ability of teachers in reading texts and in enhancing their ability to design teaching activities for real text based on context.

#### Teaching Methods

In terms of teaching methods, 6 of the teachers believe that foreign language teaching should promote the development of students’ English academic ability. Teachers will inevitably have some problems in practice, mainly manifested as follows: (1) Too much attention to testing, focusing on language training and memory, lack of design for language practice activities; (2) Basically they follow the inherent teaching model, ignoring texts and topics. As one of the teachers pointed out:

*There is still an exam baton in command, every mid-term exam, and a final exam in progress. Basically, just follow the test phases. If he does not follow, he will not get points. If you do not ask, he will not learn. Parents just want to see their test results. (T4)*

When another teacher introduced her basic teaching steps in class, she said:

*Pay attention to the pronunciation of the word, the usage of the word translated into Pinyin, the usage of the word, because the word then reaches the student’s phrase, right? The phrase, and then to the sentence to make a paragraph, a paragraph of the article is to do the whole article. If a student cannot understand the basic usage of words, he will not read the following sentence. These words are the most basic, and you must first read it by yourself. The students are in Grade two who should have read a lot; therefore, they can master them after the teacher corrected them. (T7)*

Another teacher mentioned that, due to the constraints of the learning environment, it is difficult for students to have the opportunity to learn English outside the classroom and school. Thus, it is difficult to achieve the teaching goal of developing students’ language skills.

*Quality, I feel that English, our child, after all, this environment is limited. Children can also see in class that the foundation is indeed mostly weak. In English, it is very difficult to remember words. Then there are these grammars, including reading volume, knowledge, including spoken English, and even homework. Children and even parents are relatively powerless. So, parents cannot tutor the students, and they really lack this learning for the children themselves. (T5)*

In response to the above difficulties and challenges, teachers also mentioned their needs and suggestions for teaching reforms. In summary, the development of students’ English competencies requires appropriate content in the teaching materials, development of resources, and institutional and environmental support.

Further comments from the teachers: *The content of the textbooks is too much and too difficult, causing some students to lose interest in learning English. In addition, English writing is very difficult for junior high school students, and the grammar rules are not easy to understand. (T5)*

*There should be provided more demonstrated classes or high-quality video of excellent teachers because front-line teachers are busy with classes and do not have time to listen to others’ classes. More qualified teaching resources could be produced and distributed to front-line teachers. (T7)*

*Students lack an environment of learning English at home, where parents and students are somewhat helpless. Students have mastered fewer words at the elementary school level. The foundation of the junior high school is not strong enough. The connection between elementary and secondary schools is not good enough. (T4)*

*The conditions, environment and atmosphere for learning foreign languages are not good enough, which does not attract enough attention. (T2)*

## Discussion

An innovative curriculum is essential for students to nurture, develop, and enhance their key competencies and overall competence in a foreign language, especially in English. Shu (2017) refers to the common concepts of key competencies in English as they are the key elements of education. As this has focused on the development of the key competencies for students, it should start with the teachers because they are the forerunners in imparting the reformed curriculum to the students.

Therefore, it is imperative to evaluate the knowledge of the teachers in the key competencies in English to gain a deeper insight into the potential problems in order initiate a renaissance of the curriculum. Thus, the teachers should first assimilate the cardinal concepts in the key competencies in English. It was for this reason that this research primarily focused on the ability of rural middle school teachers to implement the school-based curriculum to foster the key competencies. This underlines the necessity and importance of rural middle school teacher training. The main concern of the study is whether the teachers can distinguish between the key competencies and a sufficient understanding of the English language itself. During this investigation it was found that, in addition to theory, a more pragmatic approach will strengthen the key competencies to achieve the desired goal.

The data analysis based on the interviews of eight rural middle school teachers and the results generated from the study explicitly indicates the need for a change in teaching methods such that novel techniques are developed to teach students how to acquire the key competencies in English. Cheng (2017) reveals that “Key competencies” in China became “hot words” in 2016 with various research studies conducted in this field that paved the way for the new trend. Therefore, reviewing the research results on the key competencies in English language teaching in a timely manner will provide a useful framework for the teaching of the key competencies necessary for English language students in the future.

At this juncture, it is vital to examine why English language courses are important at the compulsory education level; the most important fact is that, at this level, the stakeholders can develop and improve their overall competence in the English language. Thus, the key competencies as researched by Wang (2011) explored the aspects beyond the level of language ability and focused on developing students’ key competencies in the English language, by examining the four aspects of language competence, cultural awareness, thinking skills, and learning ability of “Key Competence in English language education.” This research explored the various dimensions of cognition and challenges of the key competencies in the curriculum design and proposed new possibilities for further study.

In an attempt to identify the basic difficulties faced by rural middle school teachers, The Ministry of Education of the People’s Republic of China produced a study (Ministry of Education of the People’s Republic of China [MoE], 2018) which revealed that ineffective methods in classroom teaching are one of the shortcomings teachers are facing while attempting to teach the key competencies. This study also throws light upon the most crucial question which is whether a curriculum based on the key competencies-based will lead to improvements in students’ English language skills. This study shows that the teachers interviewed were capable of defining language ability and cultural behavior as a part of the key competencies. However, they were insufficiently aware of the importance of thinking skills, the need to interpret and expand dialectical thinking, which underscores an essential role in key competencies. The outcome derived from this study is quite promising and will definitely make an impact on the curriculum reforms in the teaching of the key competencies. The results show that most of the teachers are not fully equipped to introduce the practical aspects of teaching the key competency program. Some of them were not aware of the general idea behind the key competencies, although a few had a broad understanding of the need for key competencies in English. As a result of these limitations among teachers, the students are not performing well.

In this context, the studies of Mei and Wang (2018a,b) are relevant as they emphasize the concept of learning activities, which undoubtedly instill logical thinking and cooperative learning in students, and naturally improve their performance. As a result, it is essential to adopt new measures to enhance the proficiency of rural middle school teachers. Although this research studied the possibility of teacher training to reform the curriculum for enhancing key competencies, the findings suggest that the learning environment and weak foundations are the main obstacles to improving the academic proficiency of teachers in the key competencies.

The results of the study pointed toward the need to revamp existing resources, teaching materials, and institutional support. Our study has several limitations. Firstly, it only focused on the interviews of a single group of teachers. Therefore, it would be more useful if the scope of the research extended to a wider use of questionnaires and more classroom observation. Secondly, it was the study of a single school, which limits the generalization of the findings to other situations.

## Conclusion

In this article, through in-depth interviews, we surveyed eight rural junior middle school English teachers’ conceptualizations of the development of students’ key competencies in the English language, as well as the difficulties and challenges they face in the implementation of such a course. The study found that, although teachers have a positive attitude toward teaching students the key competencies of English language education, there remains a certain gap between their understanding of the key competencies and the policy requirements and the practical needs of the students. In order to implement core qualities and effectively promote curriculum reform, it is crucially important to strengthen teacher training and enhance teachers’ understanding of the curriculum requirements and strive to develop their professionalism in the new round of curriculum reform.

## Data Availability Statement

The original contributions presented in the study are included in the article/supplementary material, further inquiries can be directed to the corresponding author/s.

## Author Contributions

CJ conceived the idea for this study. FG verified the analytical methods. HL supervised the findings of this work. All authors discussed the results and contributed to the final manuscript.

## Conflict of Interest

The authors declare that the research was conducted in the absence of any commercial or financial relationships that could be construed as a potential conflict of interest.

## Publisher’s Note

All claims expressed in this article are solely those of the authors and do not necessarily represent those of their affiliated organizations, or those of the publisher, the editors and the reviewers. Any product that may be evaluated in this article, or claim that may be made by its manufacturer, is not guaranteed or endorsed by the publisher.

## Appendix

### Interview Questions

(1) What competences do you think should be cultivated in Middle School students?
(2) What do you think language ability mainly includes?
(3) What do you think language knowledge mainly includes? Do you know discourse knowledge and pragmatic knowledge?
(4) What elements do you think the English course content includes?
(5) What do you think the subject context and discourse type contain?
(6) How do you help Middle School students improve their language skills?
(7) Do you think that Middle School students’ cultural awareness can be improved through English courses?
(8) In your opinion, how to enhance Middle School students’ national feelings and global vision through English courses?
(9) What competences of thinking do you think Middle School students can improve through English courses?
(10) What learning abilities of Middle School students do you think can be improved through English courses?
(11) How do you cultivate Middle School students’ interest, habits and self-confidence in learning English?
(12) What do you think about exam-oriented education? If you agree that test-oriented education should be supported in China, why? If you oppose exam-oriented education, why? Any solution suggestions?

## References

[B1] Cheng X. Zhao S. (2016). On student key competency in English as a foreign language. *Curric. Teach. Method* 36, 79–86.

[B2] Cheng X. (2017). Key competencies of English subject and its evaluation. *Chinese Exam.* 5 7–14.

[B3] Creswell J. W. (2007). *Qualitative Inquiry and Research Design: Choosing among Five Approaches*, 3rd Edn. Thousand Oaks, CA: Sage.

[B4] Kramsch C. (2014). Teaching foreign languages in an era of globalization: introduction. *Mod. Lang. J.* 98, 296–311.

[B5] Lin C. (2012). *Psychological Anthology of Chongde Lin (China)*. Beijing: People’s Education Press.

[B6] Lin C. (2016). *Research on the Key Competencies of Student Development in the 21st Century.* Beijing: Beijing Normal University Press.

[B7] Mei D. Wang Q. (2018a). *What to Change? How to teach? How to Take the test? –Analysis of the New High School English Course Standard.* Beijing: Foreign Language Teaching and Research Press.

[B8] Mei D. Wang Q. (2018b). *Interpretation of the General High School English Curriculum Standards*, 2017 Edn. Beijing: Higher Education Press.

[B9] Ministry of Education of the People’s Republic of China [MoE] (2018). *English Curriculum Standards for General High Schools*, 2017 Edn. Beijing: People’s Education Press.

[B10] Shi J. (2014). Key competencies: in order to cultivate all-round development people. *People Educ.* 10 13–15.

[B11] Shu D. (2017). Some thoughts on the key competencies of English subject. *Shandong Foreign Lang. Teach.* 2 35–41.

[B12] Wang Q. (2011). Analysis of the policy and implementation of primary school english curriculum in China. *Chinese Foreign Lang.* 4 47–54.

